# Predictors of stable return-to-work in non-acute, non-specific spinal pain: low total prior sick-listing, high self prediction and young age. A two-year prospective cohort study

**DOI:** 10.1186/1471-2296-11-53

**Published:** 2010-07-20

**Authors:** Odd Lindell, Sven-Erik Johansson, Lars-Erik Strender

**Affiliations:** 1 Center for Family and Community Medicine, Karolinska Institutet, Alfred Nobels allé 12, SE-141 83 Huddinge, Sweden

## Abstract

**Background:**

Non-specific spinal pain (NSP), comprising back and/or neck pain, is one of the leading disorders in long-term sick-listing. During 2000-2004, 125 Swedish primary-care patients with non-acute NSP, full-time sick-listed 6 weeks-2 years, were included in a randomized controlled trial to compare a cognitive-behavioural programme with traditional primary care. This prospective cohort study is a re-assessment of the data from the randomized trial with the 2 treatment groups considered as a single cohort. The aim was to investigate which baseline variables predict a stable return-to-work during a 2-year period after baseline: objective variables from function tests, socioeconomic, subjective and/or treatment variables. Stable return-to-work was a return-to-work lasting for at least 1 month from the start of follow-up.

**Methods:**

*Stable return-to-work *was the outcome variable, the above-mentioned factors were the predictive variables in multiple-logistic regression models, one per follow-up at 6, 12, 18 and 24 months after baseline. The factors from univariate analyzes with a *p*-value of at most .10 were included. The non-significant variables were excluded stepwise to yield models comprising only significant factors (*p *< .05). As the comparatively few cases made it risky to associate certain predictors with certain time-points, we finally considered the predictors which were represented in at least 3 follow-ups. They are presented with odds ratios (OR) and 95% confidence intervals.

**Results:**

Three variables qualified, all of them represented in 3 follow-ups: *Low total prior sick-listing *(including all diagnoses) was the strongest predictor in 2 follow-ups, 18 and 24 months, OR 4.8 [1.9-12.3] and 3.8 [1.6-8.7] respectively, *High self prediction *(the patients' own belief in return-to-work) was the strongest at 12 months, OR 5.2 [1.5-17.5] and *Young age *(max 44 years) the second strongest at 18 months, OR 3.5 [1.3-9.1].

**Conclusions:**

In primary-care patients with non-acute NSP, the strong predictors of stable return-to-work were 2 socioeconomic variables, *Low total prior sick-listing *and *Young age*, and 1 subjective variable, *High self-prediction*. Objective variables from function tests and treatment variables were non-predictors. Except for *Young age*, the predictors have previously been insufficiently studied, and so our study should widen knowledge within clinical practice.

**Trial registration:**

Trial registration number for the original trial NCT00488735.

## Background

For many years, spinal pain, comprising back and/or neck pain, was the leading disorder in long-term sick-listing, including disability pensions, in Sweden as all over the industrial world. In 2002, Sweden was the leading country within the European Union in sick-listing for spinal pain [1], which in 2007 resulted in 11.9% of new disability pensions [2]. Following an international trend [3], the leading position of spinal pain in Sweden since 2005 has been overtaken by depression (in 2007 13.1% of new disability pensions) [2]. Most cases of spinal pain concern non-specific spinal pain (NSP) and are a matter for primary care [4]. In the management of disabling spinal pain, stable return-to-work is the ultimate objective [4]. As return-to-work is often followed by recurrences of work absence, longitudinal data are required to denote a stable return-to-work [5].

Cost-effectiveness in allocating treatment resources requires predictors of return-to-work to be collected by means of both questionnaires and function tests, i.e. tests in which the patient performs some kind of physical activity [6]. While the former are cheap, the latter require substantial personnel resources. Despite an immense amount of research, no gold standard for questionnaires and/or tests has been established for this purpose [6,7]. In the treatment of non-acute NSP, i.e. pain leading to full-time sick-listing for more than 3 weeks [8], evidence-based guidelines advocate a cognitive-behavioural therapeutic approach [4].

During 2000-2004, 125 patients with non-acute NSP were included in a randomized, controlled trial to compare a cognitive-behavioural programme with traditional primary care [9]. A package of function tests and a questionnaire were completed at baseline. The aim of this study was to answer the question "which are the predictors at baseline in non-acute NSP for stable return-to-work during a 2-year period after baseline: objective variables from function tests, socio-economic, subjective and/or treatment variables?"

## Methods

### On sick-listing and return-to-work in Sweden

As the employer has the financial responsibility for the 2 initial weeks of sick-listing in Sweden, the available data include only the sick-listing periods exceeding 2 weeks. For the unemployed subjects, however, those data include all periods. Sick-listing, as described in detail in a prior study [9], might have the degrees .25, .50, .75 or 1.00 (= full-time). The degree of return-to-work = 1.00 minus the degree of sick-listing, as defined by the Social Insurance Agency. For example, sick-listing = .75 equals return-to-work = .25 and full-time sick-listing equals non-return-to-work. In response to prolonged sick-listing, the Agency might consider a temporary or permanent disability pension (the temporary form being abolished in 2008), which might have the same degrees as the other forms of sick-listing.

### Setting and source population

The setting was a suburban area in the Southern part of Stockholm County, including 9 municipalities with a population of 466,000, of whom 288,000 of working age (18-64 years) constituted the source population.

### Patients

One hundred and twenty-five primary-care patients with non-acute NSP were recruited to a randomized controlled trial, which in detail was described in a previous study [9], by 41 family doctors at 13 health centres between August 2000 and January 2004. Recruitment was non-systematic, i.e. it was up to the family doctor on the basis of her or his current motivation and available time to invite a potentially eligible patient. In summary:

The patients were allocated either to a cognitive-behavioural programme at a rehabilitation centre or continued traditional primary care. *The criteria for inclusion: *1.Vocationally active, up to and including 59 years of age. 2. Sick-listed full-time for spinal pain at least six weeks (42 days) and at most two years (730 days). 3. Able to fill in forms. *The criteria for exclusion: *1. Temporary disability pension or disability pension being paid or in preparation. 2. Primary need for action by a hospital specialist (for example, operation for intervertebral herniation (slipped disc)). 3. Pregnancy or diseases (other than spinal pain) that might make the rehabilitation programme impracticable (for example, advanced pulmonary disease). 4. Whiplash-associated disorders as a primary obstacle to working. 5. Previous rehabilitation at the rehabilitation centre. 6. Other multidisciplinary rehabilitation measures ongoing or planned.

The recruited patients were interviewed by telephone by a research assistant within 2 days. The patients who remained qualified saw the assistant at the health centre within 5 days. Before the assistant carried out the randomization, certain procedures were completed: the patient finished a questionnaire, including a pain drawing; the assistant categorized the pain as being back and/or neck pain, basing the decision on how the patient completed the pain drawing and by a short interview. The back was taken as the area below an imaginary line connecting the lower tips of the shoulder blades, including the lower half of the thoracic spine and the lumbar spine; and the neck was the area on and above this line, including the upper half of the thoracic spine and the cervical spine [10]; the patient also performed a package of 10 function tests as described in detail in a previous study [11].

### Design

This prospective cohort study is a re-assessment of the data from the randomized controlled trial with the 2 treatment groups considered as a single cohort.

#### Outcome variable

##### Stable return-to-work

The outcome variable was *Stable return-to-work*, which required that a return-to-work on a specific day lasted for at least 1 month. For example, a *Stable return-to-work *on 6 June required that the return-to-work continued at least up to and including 5 July. The reference to *Stable return-to-work *was *Non-return-to-work*, including non-return-to-work a specific day and return-to-work that day but with recurrence of work absence the following month. Due to the responsibility of the employer, *Stable return-to-work *possibly contained a period of work absence of a maximum of 14 days during the follow-up month including the specific day. *Stable return-to-work *was analyzed in 4 specific days during a 2-year period, selected as 6, 12, 18 and 24 months after baseline.

#### Predictive variables

##### Objective variables

Six reliable function tests from the 10-test package were used as objective variables. In a previous study, we had examined the reliability, including inter- and intra-rater reliability, of the package [11]. In summary, 2 examiners participated, an experienced physiotherapist and a research assistant. All the 5 tests that did not require manual fixation of the patient by the examiner were reliable. Only 1 of the 5 tests which required fixation was reliable. In conclusion, 6 of the 10 tests were reliable and could be used by an examiner lacking formal medical education (the research assistant) without loss of quality. Two of those tests included flexion to the right and to the left and rotation to the right and to the left, and a lift test comprised a lumbar and a cervical subtest. Nine subtests in total are given in Table 1.

**Table 1:** Objective variables. Results of univariate-logistic regression, adjusted for gender and age, with *p*-values of at most .10.

			**Prediction for *Stable return-to-work***											
			
			**6 months**			**12 months**			**18 months**			**24 months**		
						
**Subtests**	**Class limits**	**n**	**OR**	***p***	**95%CI**	**OR**	***p***	**95%CI**	**OR**	***p***	**95%CI**	**OR**	***p***	**95%CI**
				
*Forward flexion *(centimeters (cm))	25-64	41							Ref.			Ref.		
	8-24	42	-	-	-	-	-	-	3.4	.01	1.3-8.8	2.6	.05	1.0-6.5
	0-7	41	-	-	-	-	-	-	2.1	NS	.8-5.6	1.3	NS	.5-3.2
				
*Modified Schober *(cm)	1-3	18												
	4-6	83	-	-	-	-	-	-	-	-	-	-	-	-
	7-19	23	-	-	-	-	-	-	-	-	-	-	-	-
				
*Lateral flexion right *(cm)	3-10	41							Ref.					
	11-15	39	-	-	-	-	-	-	2.3	.09	.9-6.2	-	-	-
	16-28	44	-	-	-	-	-	-	1.9	NS	.8-4.9	-	-	-
				
*Lateral flexion left *(cm)	2-11	41							Ref.					
	12-15	38	-	-	-	-	-	-	2.9	.03	1.1-7.6	-	-	-
	16-27	45	-	-	-	-	-	-	1.8	NS	.7-4.7	-	-	-
				
*Cervical rotation right *(degrees)	0-50	44										Ref.		
	51-60	43	-	-	-	-	-	-	-	-	-	2.6	.04	1.0-6.6
	61-80	37	-	-	-	-	-	-	-	-	-	2.7	.05	1.0-7.1
				
*Cervical rotation left *(degrees)	0-50	47												
	51-60	39	-	-	-	-	-	-	-	-	-	-	-	-
	61-80	38	-	-	-	-	-	-	-	-	-	-	-	-
				
*Abdominal endurance *(seconds)	0	46												
	1-14	40	-	-	-	-	-	-	-	-	-	-	-	-
	15-75	38	-	-	-	-	-	-	-	-	-	-	-	-
				
*PILE lumbar *(kilogram)	0-6	33												
	8-12	45	-	-	-	-	-	-	-	-	-	-	-	-
	14-44	46	-	-	-	-	-	-	-	-	-	-	-	-
				
*PILE cervical *(kilogram)	0-6	37	Ref.						Ref.					
	8-12	47	1.4	NS	.5-4.4	-	-	-	1.1	NS	.4-2.9	-	-	-
	14-44	40	2.9	.09	.9-9.5	-	-	-	2.8	.06	1.0-8.4	-	-	-

OR = Odds ratio. 95%CI = 95% confidence interval. Ref. = Reference, which always has OR = 1.0. NS = Non-significant (*p *> .10).

The rationales for the choice of the function tests were established in a previous study [11].

#### Socioeconomic variables

These were collected from the questionnaire except data for the 2 sick-listing variables, which were collected from The Social Insurance Office. The sick-listing variables were: *Subacute NSP *= current, full-time sick-listing at baseline for NSP of 6-12 weeks (42-84 days) with the reference *Chronic NSP *= current, full-time sick-listing of more than 12 weeks up to and including 2 years (85-730 days) [8], and *Low total prior sick-listing *= at most 183 net days during the 2 years prior to baseline, including all diagnoses, with the reference *High total prior sick-listing *≥ 184 net days [12]. 'Net days' is the sick-listing expressed in whole days = crude days × degree [13]. In total, 23 socioeconomic variables are presented in Table 2.

**Table 2:** Socioeconomic variables.

		**Prediction for *Stable return-to-work***											
		
		**6 months**			**12 months**			**18 months**			**24 months**		
					
	**n**	**OR**	***p***	**95%CI**	**OR**	***p***	**95%CI**	**OR**	***p***	**95%CI**	**OR**	***p***	**95%CI**
				
*Man *[24,27,29,34]	56	-	-	-	2.1	.06	1.0-4.5	-	-	-	-	-	-
				
*Young age *(≤44 years)[26,30]	67	-	-	-	3.0	.005	1.4-6.7	2.9	.006	1.3-6.1	2.6	.01	1.2-5.4
				
*Non-immigrant*[12]^1^	90	-	-	-	-	-	-	-	-	-	-	-	-
				
*Co-habiting*[53]^2^	85	-	-	-	-	-	-	-	-	-	-	-	-
				
*Living without children*[54]	55	-	-	-	-	-	-	-	-	-	-	-	-
				
*Non-bad economy*[17]^3 ^	68	-	-	-	-	-	-	-	-	-	-	-	-
				
*Non-low education*[55]^4^	80	2.2	.07	.9- 5.6	2.9	.02	1.2-7.1	3.0	.01	1.3-6.9	3.5	.004	1.5-8.0
				
*White-collar job*[56]^5^	12^5^	-	-	-	-	-	-	-	-	-	-	-	-
				
Physical work conditions^6^:													
*No vibrations*[58]	84	3.3	.03	1.2-9.4	2.9	.04	1.0-7.0	-	-	-	-	-	-
*Light physical workload*[34]	21	-	-	-	-	-	-	-	-	-	-	-	-
*Varied work moments*[34]	46	-	-	-	-	-	-	-	-	-	-	-	-
*Non-sedentary work*[57]	88	-	-	-	-	-	-	-	-	-	-	-	-
*Comfortable w. postures*[34]	27	-	-	-	-	-	-	-	-	-	-	-	-
				
Psychosocial work conditions^7^:													
*No job strain*[60]	90	-	-	-	-	-	-	-	-	-	-	-	-
*Good social support*[61]	94	4.5	.02	1.2-16.2	-	-	-	-	-	-	2.7	.04	1.1-6.8
				
*Non-unemployed*[62]	95	.5	.10	.2-1.2	-	-	-	-	-	-	-	-	-
				
*No work trauma litigation *^8^	97^8^	-	-	-	-	-	-	-	-	-	-	-	-
				
*Non-smoking*[26]	75	-	-	-	-	-	-	-	-	-	-	-	-
				
*No indication of alcohol overconsumption*[42]^9^	107	-	-	-	-	-	-	-	-	-	-	-	-
				
*High physical activity*[65]^10^	86	-	-	-	-	-	-	-	-	-	-	-	-
				
*Non-obese *(BMI < 30[66])[37]	94	.4	.05	.2-1.0	-	-	-	-	-	-	-	-	-
				
*Subacute NSP *^11^	38	3.4	.006	1.4-8.0	2.8	.02	1.2-6.3	5.4	< .001	2.2-13.0	3.1	.008	1.4-7.2
				
*Low total prior sick-listing *^12 ^	57	3.1	.005	1.4-6.9	3.1	.005	1.4-6.9	7.7	< .001	3.3-18.1	4.9	<.001	2.2-11.0

^1 ^= Born in Sweden. **Reference: ***Immigrant *(n = 34).

^2 ^Includes living single with children. **Reference: ***Single *= living alone, without children (n = 39).

^3 ^= "Neither bad nor good", "Good" or "Very good". **Reference: ***Bad economy *= "Very bad" or "Bad" (n = 56).

^4 ^**Reference: ***Low education *= at most junior high school (n = 44).

^5 ^Out of the 94 non-unemployed patients. **Reference: ***Blue-collar job *(n = 82).

^6 ^"State the conditions that you regularly (not occasionally) are exposed to: ...Vibrations (from tools, vehicles etc.) ...Heavy lifting or greater muscle efforts ...Monotonous work moments ...Sedentary work ...Difficult work postures (bent, twisted, locked etc.)": answer "No". **References: **"Yes" [57].

^7 ^Psychological demands (5 items), decision latitude (6 items) and social support (6 items), total scores 5-20, 6-24 and 6-24 respectively; *No job strain *= non-scoring demands above the midpoint (> 13) and decision latitude below the midpoint (> 15); **reference: ***Job strain *= demands above + decision latitude below the midpoint (n = 34). *Good social support *= above the midpoint (> 15); **reference: ***Bad social support *= below the midpoint (n = 30) [59].

^8 ^Out of 115 patients (9 patients scored "I don't know"). "Have you reported your pain as a work trauma?": answer "No". **Reference: **"Yes" (n = 18) [63].

^9 ^To drink alcohol corresponding to at least 1/2 bottle (= 37.5 centilitres) of strong spirits on one and the same occasion, less than 2-3 times monthly. **Reference: ***Indication of alcohol overconsumption *= at least 2-3 times monthly (n = 17) [64](+ personal communication Anders Romelsjö 27 Aug. 2007).

^10 ^Physical activity, including walking > 30 minutes, twice/week or more. **Reference: ***Low physical activity*: once/week or less (n = 38).

^11 ^= a current, full-time sick-listing at baseline for NSP 42-84 days. **Reference: ***Chronic NSP *= a corresponding sick-listing of 85-730 days (n = 84) [31].

^12 ^= a prior 2-year sick-listing for all diagnoses of at most 183 net days. **Reference: ***High total prior sick-listing *≥ 184 net days [12].

Further explanations in Table 1.

#### Subjective variables

Except for the division into back and/or neck pain, the subjective variables were collected exclusively from the questionnaire. They included different aspects of pain, mental mood, comorbidity, loss of function due to NSP, health-related-quality of life, coping with pain, and a question about the probability of return-to-work: "What do you believe, honestly, is the probability that you will become so much better that you will be able to work at some time in the future?" [14]. *High self prediction *included the answering alternatives 'rather probable','probable' and 'very probable', and *Low self prediction *the alternatives 'rather improbable', 'improbable' and 'very improbable'. A similar type of question was used by Linton et al. [15], but included a future time-limit of 6 months, i.e. a much shorter period than our 2-year follow-up. We therefore chose the open-ended question from Eklund et al. [14]. A total of 16 subjective variables are shown in Table 3.

**Table 3:** Subjective variables.

			**Prediction for *Stable return-to-work***											
			
			**6 months**			**12 months**			**18 months**			**24 months**		
						
	**Class limits**	**n**	**OR**	***p***	**95%CI**	**OR**	***p***	**95%CI**	**OR**	***p***	**95%CI**	**OR**	***p***	**95%CI**
				
*Pain just now *(VAS 1-100)[25]	70-100	41	Ref.											
	48-69	43	2.4	.09	.9-6.9	-	-	-	-	-	-	-	-	-
	0-47	40	1.5	NS	.5-4.3	-	-	-	-	-	-	-	-	-
				
*Pain at worst last week*[25]	81-100	42	Ref.											
	68-80	43	2.5	.09	.9-6.8	-	-	-	-	-	-	-	-	-
	0-67	39	1.4	NS	.5-4.2	-	-	-	-	-	-	-	-	-
				
*Intermittent pain*[15]^1^	-	39	-	-	-	-	-	-	2.3	.04	1.0-5.4	-	-	-
				
*Non-radiating pain*[17]^2^	-	32	-	-	-	-	-	-	-	-	-	-	-	-
				
*Local pain*[25]^3^	-	24	-	-	-	-	-	-	-	-	-	-	-	-
				
*Back-pain domination*[32]^4 ^	-	86	9.0	.004	2.0-40.2	2.5	.05	1.0-6.4	-	-	-	-	-	-
				
*Time since start of NSP *(years)[27]	> 5	53							Ref.			Ref.		
	1.5-5	34	-	-	-	-	-	-	2.9	.03	1.1-7.4	2.2	.09	.9-5.5
	< 1.5	37	-	-	-	-	-	-	1.5	NS	.6-3.6	1.1	NS	.5-2.7
				
*No surgery f. b/n pain*[50]^5^	-	116	-	-	-	-	-	-	-	-	-	-	-	-
				
*No anxiety/depression*[15]^6^	-	26	-	-	-	-	-	-	-	-	-	-	-	-
				
*Tired seldom*[67]^7^	-	59	3.1	.01	1.3-7.6	-	-	-	1.9	.09	.9-4.2	-	-	-
				
*No comorbidity*[68]^8^	-	79	-	-	-	-	-	-	-	-	-	-	-	-
				
*Non-severe functional impairment *(ODI)^9^	-	78	2.1	.09	.9-4.9	2.9	.01	1.3-6.8	2.5	.02	1.2-5.4	-	-	-
				
*Health-related*	0-.359	42	Ref.			Ref.			Ref.					
*quality of life*	.360-.629	46	2.8	.06	1.0-8.3	2.1	NS	.8-5.4	2.1	NS	.8-5.1	-	-	-
(EQ-5D)[21]	.630-1.0	36	2.9	.06	.9-8.9	2.6	.06	1.0-7.1	3.0	.03	1.1-7.9	-	-	-
				
*State of health *(EQ-VAS)[21]	0-35	44	Ref.			.			Ref.					
	36-49	33	2.2	NS	.7-7.0	-	-	-	2.0	NS	.7-5.4	-	-	-
	50-100	47	3.6	.02	1.3-10.3	-	-	-	3.1	.01	1.3-7.7	-	-	-
				
*Non-catastrophizing*[70]^10 ^	-	67	2.2	.08	.9-5.1	-	-	-	3.6	.002	1.6-8.0	2.3	.04	1.1-4.9
				
*High self prediction*[14]	-	95	4.2	.03	1.2-15.2	6.4	.002	1.9-21.0	4.4	.005	1.5-12.4	3.8	.008	1.4-10.2

^1 ^**Reference: ***Continual pain *= pain whenever awake (n = 95).

^2 ^**Reference: ***Radiating pain *= radiation of pain/numbness to the leg beneath the knee and/or the arm beneath the elbow (n = 92).

^3 ^Pain in the back or the neck. **Reference: ***Widespread pain *= pain in both the back and the neck (n = 100).

^4 ^**Reference: ***Neck-pain domination *(n = 38).

^5 ^**Reference: **Surgery for back and/or neck pain at least once (for example, for a slipped disc) (n = 8).

^6 ^Item 5 in EQ-5 D[20], alternative 1 = "I am not anxious or depressed". **Reference: **alternative 2: "... moderately..." or 3: "... extremely...".

^7 ^One item from SF 36[67]: "Tired during the last four weeks: 'some of the time', 'a little bit of the time' or 'none of the time'". **Reference: ***Tired often = *'all of the time', 'most of the time' or 'a good bit of the time'.

^8 ^**Reference: ***Comorbidity *= any other, chronic disease except NSP or obesity (n = 45).

^9 ^ODI (Oswestry Disability Index) scores general functional disability associated with back pain, 0-100%: 0-20% = minimal, 21-40% = moderate, 41-60% = severe, 61-100% = extremely severe to crippling disability[38]. **Reference: ***Non-severe functional impairment *= ODI < 41%[69]

^10 ^Six catastrophizing thoughts, never-always, 0-6, are summarized, 0-36. *Non-catastrophizing *≥ 15. **Reference: ***Catastrophizing *> 15 (n = 39).

Further explanations in Table 1.

#### Treatment variables

Sixty-three of the 125 patients received *Cognitive-behavioural rehabilitation *and 62 patients received the reference treatment of *Traditional primary care*. The treatment options were described in detail in a previous study [9].

### Statistics

STATA10.1 was used for the calculations [16].

### Power calculation

The power calculation of the randomized controlled trial has been described in a previous study [9]. In this prospective cohort study we were reduced to analyze the number of patients who were already included in the randomized controlled trial. However, several prior prediction studies included a comparable number of patients, e.g. Eklund et al. [14] 149 patients, Lancourt et al. [17] 134 patients, and Linton et al. [15] 142 patients.

#### Stable return-to-work

*Stable return-to-work *for 6, 12, 18 and 24 months, and of disability pension in 24 months were calculated. The proportions were compared between the genders by univariate-logistic regression, adjusted for age (*Young age *= 18-44 years and *Older age *= 45-59 years) and are given with *p*-values [18]. In the logistic regression *Stable return-to-work *might have the values 1, including the degrees .25, .50, .75 and 1.00, or 0 = *Non-return-to-work*.

### Multiple-logistic regression

We built 4 multiple-logistic regression models for each of the follow-ups at 6, 12, 18 and 24 months. The outcome variable was *Stable return-to-work*. The predictive variables were the above described objective, socioeconomic, subjective and treatment variables. Ordinal and continuous variables were divided into classes. The models were adjusted for gender and age. We first explored univariate analyses. The variables with a *p*-value of at most .10 are presented with odds ratios (OR), *p*-values and 95% confidence intervals (CI). They were included in a multiple model, from which the variables with *p*-values of .05 or higher were excluded step-wise to yield a model comprising only variables with *p*-values < .05. However, in the choice between a model with a larger number of variables including those with *p*-values of .05 or slightly above and a smaller model with *p*-values exclusively smaller than .05, the larger model was tested against the smaller model (STATA commandos "estimates store full" and "lrtest full"). If that test produced a *p*-value smaller than .05, the larger model was chosen as the ultimate one, otherwise the smaller model [18]. All possible first-order interaction terms were tested in each model.

Although it is important that a multiple-logistic regression model includes all relevant predictor variables, it is also important that the model does not include more predictors than the given number of observations justify. The existence of sufficient events per variable was emphasized by Bagley et al. [19] in a large overview of logistic regression. The number of the less common of 2 possible outcomes (in our study *Stable return-to-work *or *Non-return-to-work*) divided by the number of predictor variables was recommended to be at least 10 and preferably more [20]. On the basis of the number of patients with *Stable return-to-work *(Table 4), the maximal possible numbers of predictors were calculated as 3, 5, 5-6 and 6 at 6, 12, 18 and 24 months, respectively. While the models of 18 and 24 months lived up to that with 5 and 4 variables each, the models of 6 and 12 months included 5 and 6 variables, which necessitated the exclusion of 2 and 1 predictors respectively. We excluded from the 6-month model *No vibrations*, OR 5.9 [1.7-20.8](95% CI), *p *= .006, since this variable was represented in only one of the other follow-ups; and *Tired seldom*, OR 3.3 [1.2-9.4], *p *= .02, since it was not found in other follow-ups. From the 12-month model *No vibrations*, OR 3.2 [1.1-9.3], *p *= .03, was excluded, since it was found in only one of the other follow-ups. By the exclusion of *No vibrations *one of the remaining variables, *Man*, became non-significant (*p *changed from .02 to .08), and was left outside the final presentation of the 12-month model. Like *No vibrations*, *Back-pain domination *was represented in only the 6- and 12-month follow-ups, but was retained in the models due to its outstanding OR in 6 months. The results of the final models are shown as OR with *p*-values and 95% CI with goodness-of-fit tests by Hosmer-Lemeshow, the percentages of correctly predicted patients and the areas under ROC-curves [18].

**Table 4:** *Stable return-to-work*.

	**6 months**			**12 months**			**18 months**			**24 months**		
				
	**n**	***Stable return-to-work*** **n (%)**	***p***	**n**	***Stable return-to-work*** **n (%)**	***p***	**n**	***Stable return-to-work*** **n (%)**	***p***	**n**	***Stable return-to-work*** **n (%)**	***p***
				
All	124	33 (26.6)	-	123	48 (39.0)	-	122	55 (45.1)	-	122	58 (47.5)	-
				
Men	56	19 (33.9)	NS	55	27 (49.1)	NS	54	29 (53.7)	NS	54	30 (55.6)	NS
								
Women	68	14 (20.6)	NS	68	21 (30.9)	NS	68	26 (38.2)	NS	68	28 (41.2)	NS

NS = Non-significant (*p *≥ .05).

All patients and gender. The proportions are compared by univariate logistic regression, adjusted for age.

We found no comparable studies of return-to-work at several time-points; for example, Hansson et al. [21] analyzed return-to-work at 90 days, 12 and 24 months, but their study included ~1500 subjects and no objective variables. However, we appraised that the comparatively small number of cases in our study made it risky to associate certain predictors with certain time-points. We chose to take into final consideration only the variables that were represented in at least 3 of the 4 follow-ups.

### Ethical approval

Approval for the study was given by The Research Ethics Committee, Karolinska University Hospital, Huddinge.

## Results

**Figure 1:**
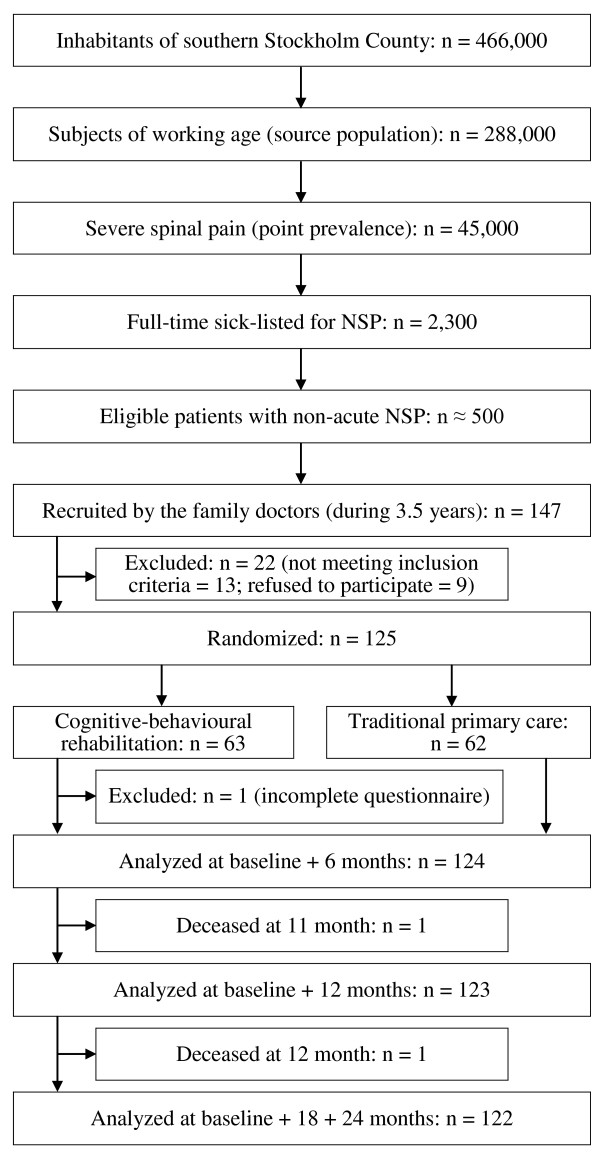
**Flowchart**. Further explanation can be found in the text.

A flow-chart of the study is shown in Figure 1.

### Source population

From data in a cross-sectional study under preparation, the point prevalence of severe spinal pain in the source population was estimated at 15.6% or ~45,000 subjects, and of full-time sick-listing due to spinal pain to .8% or ~2,300 subjects, including short- and long-term sick-listing. The data were collected from Statistics Sweden, a governmental authority [22]. The great majority of patients with disabling NSP recovers quickly. Roughly, after full-time sick-listing 1 week around 50% and after 12 weeks 90% of the patients have returned to work. Thereafter the recovering speed evidently levels off [8]. We estimated the point prevalence of non-acute NSP in the source population to around .2% or 500 subjects. We have no data of the prevalence over time.

### Loss to follow-up

Three of the 125 patients, all males, deceased during the follow-up, 11, 12 and 22 months after baseline. The last deceased patient was excluded from the study because of an incomplete questionnaire. The other 2 subjects were analyzed up to their possible follow-ups. The questionnaires of 124 patients were analyzed and sick-listing data were collected at 6, 12, 18 and 24 months for 124, 123, 122 and 122 patients, respectively.

### Study population

Of the 124 patients, *Subacute NSP *and *Chronic NSP *occurred in 38 (30.6%) and 86 (69.4%) patients, respectively. The current sick-listing period at baseline was *m *172 [149-194], days. *Back-pain domination *and *Neck-pain domination *was seen in 86 (69.4%) and 38 (30.6%) patients, respectively. Twenty-four patients (19.4%) had *Local pain*, i.e. back or neck pain, and 100 patients (80.6%) had *Widespread pain*, i.e. both back and neck pain.

#### Stable return-to-work

*Stable return-to-work *gradually increased and was 58/122 (47.5%) at 24 months, a majority at full-time (43/58 = 74.1%). The proportions were generally higher for men, but the gender differences were non-significant (Table 4). At 24 months, disability pension (temporary or permanent) was received by 30/122 (22 full- and 8 half-time pensions), with a significantly higher proportion of women, 22/68 (32.4%), than men, 8/54 (14.8%) (*p *= .04).

### Predictors of Stable return-to-work

In the univariate analyses, several objective, socioeconomic and subjective variables were associated with *Stable return-to-work *(Tables 1, 2, 3, while the treatment variables, *Cognitive-behavioural rehabilitation *or *Traditional primary care*, were not predictive in any of the follow-ups.

In the multiple-logistic models only socioeconomic and subjective variables remained, of which 3 variables were finally considered, all of them represented in 3 follow-ups (Table 5): *Low total prior sick-listing*, including all diagnoses, was the strongest predictor in 2 follow-ups, and *High self prediction *and *Young age *were the strongest and second strongest, respectively, in one of the follow-ups. In the models there were 3, 6, 10 and 10 first-order interaction terms, respectively, but none was predictive. The model fit was generally good and the proportions of correctly classified patients were satisfactory (on average 74.1%).

**Table 5:** Predictors of *Stable return-to-work*.

	**Prediction for *Stable return-to-work***											
	
	**6 months**			**12 months**			**18 months**			**24 months**		
				
	**OR**	***p***	**95%CI**	**OR**	***p***	**95%CI**	**OR**	***p***	**95%CI**	**OR**	***p***	**95%CI**
				
***Young age***	-	-	-	**2.8**	**.02**	**1.2-6.5**	**3.5**	**.001**	**1.3-9.1**	**2.7**	**.02**	**1.2-6.2**
*Non-low education*	-	-	-	-	-	-	3.0	.04	1.1-8.2	2.9	.02	1.2-6.9
*Subacute NSP*	3.2	.02	1.3-8.2	-	-	-	3.0	.04	1.1-8.4	-	-	-
***Low total prior sick-listing***	-	-	-	**2.7**	**.02**	**1.2-6.4**	**4.8**	**.001**	**1.9-12.3**	**3.8**	**.002**	**1.6-8.7**
*Back-pain domination*	9.5	.004	2.0-44.4	2.9	.04	1.1-7.7	-	-	-	-	-	-
*Non-catastrophizing*	-	-	-	-	-		3.4	.01	1.3-9.1	-	-	-
***High self prediction***	**4.1**	**.02**	**1.1-15.7**	**5.2**	**.009**	**1.5-17.5**	-	-	-	**2.7**	**.06**	**.9-7.8**
				
Goodness-of-fit:												
Hosmer-Lemeshow	.70			.38			.29			.67		
Correctly classified (%)	78.2			71.5			73.0			73.8		
Area under ROC	.79			.79			.85			.79		

Multiple-logistic regression. The variables found in at least three follow-ups are in bold text.

## Discussion

The predictors of stable return-to-work were analyzed in 124 patients with non-acute NSP. Of the total of 50 variables, 2 socioeconomic variables, *Low total prior sick-listing *and *Young age*, and 1 subjective variable, *High self prediction*, were finally considered. None of the objective variables from function tests and of the treatment variables were predictive.

### Predictors in the study compared with prior research

*Young age *is in line with several previous studies and reviews [1,23-27]. Also *High self prediction *is a well known predictor [14,15,28,29]. For example, the basic question in the clinical algorithm for return-to-work prediction by Dionne et al. [28] concerned the patient's own recovery expectations.

One of the most consistent predictors in previous research was low prior sick-listing for spinal pain [26,30,31]. While *Subacute NSP *was one of the strongest predictors in the univariate analyses, it was outflanked in the multiple context by *Low total prior sick-listing*, except at 6 months. According to one of the hitherto most extensive reviews of predictors of long-term sick-listing for spinal pain, prior sick-listing for all diagnoses has been insufficiently studied [1]. Our results indicate that it is very important to map prior sick-listing for all diagnoses, not only for spinal pain. This is also in line with the Örebro Musculoskeletal Pain Questionnaire, a widely-used screening instrument [15], and with fairly recent studies with prolonged follow-ups [12,32].

### Non-predictors in the study compared with prior research

Two non-predictors were in line with previous studies, *Comfortable work postures *[26,30] and *Good social support *[25,26]. *Non-Smoking *as a non-predictor is supported by some studies [33,34], but is contradicted by others: a large review of mostly cross-sectional studies indicated a possible association between NSP and cigarette smoking, but emphasized the lack of prospective studies [35]. Recent, prospective studies pinpointed cigarette smoking as a strong predictor of non-return-to-work in men, Dionne et al. [5], and as a moderate predictor of non-return in both sexes, Stillgate et al. [36].

Six of our non-predictors contradicted prior research:

*Man *and *Non-low education *were non-predictors, while prior research indicated them as predictors, at least for disability pension [1,12]. However, the proportion of disability pension at 24 months was significantly lower for men and *Non-low education *was close to qualify with a representation at both 18 and 24 months. It is logical that a disability pension will be granted only after prolonged sick-listing and that education might influence return-to-work comparatively late in a rehabilitation process, when the medical efforts have been replaced by vocational measures. Consequently, our findings might be in line with prior research, although a longer follow-up than 2 years is required to confirm this.

*High physical workload*, the reference to *Light physical workload*, is a well-established predictor of low return-to-work [26,30,34,37], but was non-predictive in our study. The large majority of our patients (83.2%) had a *High physical workload *(compared to 15.4% of a population-based local sample in a cross-sectional study in preparation [22]). Thus, a variable of such overwhelming frequency might be non-discriminative, although it has a powerful effect on sick-listing.

*Non-severe functional impairment*, as measured by the Oswestry Disability Index [38-40], *Health-related quality of life*, according to EQ-5 D [21,41], and *State of health*, as expressed by EQ VAS [21], were comparatively strong predictors in the univariate analyses, but non-predictors in the final multiple-logistic models. This is contrary to previous studies [5,21,25,38-40], for which we can offer no explanation.

### Non-predictors in the study that have previously been insufficiently studied

Many of our non-predictors that have been insufficiently studied in previous research might contribute to a widening of knowledge: *Non-immigrant, Co-habiting*, *Living without children*, *Non-unemployment, No work trauma litigation, Non-bad economy, Non-obese*, *No comorbidity*, *No surgery for spinal pain, Pain duration, Pain intensity, Local pain, Back-pain domination, High physical activity, Varied work moments, No job strain, No depression/anxiety *and *No indications of alcohol over-consumption *[1].

Concerning pain localisation and alcohol, prior studies are conflicting: While the predictive value of spinal pain localization has been questioned [1,15], recent research, including very large samples, supports the positive effect on return-to-work of *Back- *versus *Neck-pain domination *[21,32]. *Back-pain domination *in our study was near to qualify with a strong representation in 2 follow-ups. So, the non-prediction might be due to the comparatively low number of patients. While one study showed no association between alcohol over-consumption and sick-listing for spinal pain [41], another study found that alcohol abuse was higher among persons with chronic spinal pain [42]. A recent large study indicated that moderate alcohol consumption tended to decrease sick-listing for NSP, at least among women in the public sector [36].

### Objective versus subjective variables

As few of the function tests commonly used in previous research were validated, it is difficult to judge from prior studies if objective variables are predictive [6]. For example, in a Cochrane review of specific spinal pain, subjective variables such as the state of health predicted return-to-work, but there was insufficient scientific support concerning objective variables, such as strength or motion range [7]. Our study strongly supports the predictive value of subjective predictors and might widen the knowledge of objective variables as non-predictors.

### Treatment as a predictor of return-to-work

For the entire group of patients, treatment was non-predictive. In a previous study [9], there were indications that patients with *Subacute NSP *had a greater return-to-work chance when they received the cognitive-behavioural programme. However, a more detailed evaluation of the possible positive effect on return-to-work of our programme requires other analyses than in the present study - for example, survival analysis as in the previous study [9] - and is a matter for future work.

### Strengths of the study

The prospective design, with a comparatively long follow-up period, is a major strength of our study.

The generalisation of the results of previous research on the prediction of return-to-work in spinal pain is seriously limited by the under-representation of women [1]. Thus one strength of our study has been the good representation of women.

We have no data of the proportion of work obstacles due to back pain compared with neck pain in the source population. In previous research, the annual prevalence in industrial countries of work obstacles due to back pain and neck pain has been estimated to 8% and 2%, respectively [43]. We obtained a similar ratio, which might indicate that our study sample is representative of subjects with non-acute NSP.

Because we used data from the Social Insurance Office, no sick-listing data was missing, except the possible short-term relapses of non-return-to-work during the follow-up months. With the exclusion of one patient, the questionnaire data were complete.

The use of reliable function tests is a major strength. One of the examiners in our reliability study [11], the research assistant, also carried out the function tests in this study.

### Limitations of the study

Some circumstances might have decreased the representativeness of the study sample, and increased the risk of bias. The above-mentioned annual prevalence of work limiting back pain and neck pain corresponds to ~23,000 and ~5,000, respectively, in the source population. Though these data include short-term sick-listing also, it is obvious that the study population of 125 patients recruited over a period of 3.5 years constituted a very low percentage of the eligible subjects. As a comparison, Dionne et al al. [28] achieved a participation rate of 68.4% of eligible subjects. The inclusion was non-systematic: a family doctor with a local reputation of great skills in spinal pain might attract more complex cases, and have a higher motivation for research and the recruitment of study patients. This might lead to spectrum bias, i.e. the effect the patient mix may have on the performance of tests, e.g., a package of predictors [44]. We were overoptimistic concerning the recruiting propensity of the family doctors and lacked resources to make them more compliant. This contributed to a prolonged inclusion period (3.5 years) that increased the probability of societal changes in rules and attitudes concerning sick-listing and might result in different return-to-work predictors in identical spinal pain due to inclusion either early or late in the recruitment period. The problem with protracted inclusion periods is shared with several other studies. For example, Lindström et al. [45] and Loisel et al. [46] used 2.5 years for the inclusion of 103 and 130 patients, respectively, and Jensen et al. [47] 3.5 years for 214 patients. As a comparison, Dionne et al. [28] used a systematic approach and recruited 1007 patients in about 1.5 years.

While it is advocated that predictive conclusions might be drawn exclusively from studies with a sick-listing-baseline on day zero [48], our patients had been sick-listed for at least 6 weeks at baseline, which might be seen as a limitation. However, even in the above-mentioned large review [1], several of the studies had baselines similar to ours [17,26,34] and arguably it is also of great interest to predict return-to-work in non-acute NSP.

Work satisfaction as a separate variable was not included. Since work satisfaction was indicated as a return-to-work predictor in several previous studies [25,49,50], it is a limitation.

There is no gold standard enabling the analysis of the time-points of return-to-work [51], but logically different predictors have a different impact in different time-points. While education might have a stronger influence comparatively late, pain and other subjective variables might affect the outcome early. It is also of great interest to know what variables predict return-to-work and when. For example, prediction of return-to-work, but not until 24 months, might be of no use concerning a patient close to old-age pension. A limitation of our study is that the follow-ups are not mutually compared, which should require a larger number of cases.

As cognitive-behavioural therapy, among other items, addresses dysfunctional beliefs [52], *Cognitive-behavioural rehabilitation *given to half of the patients might have a greater impact on the self prediction and result in an underestimation in the association between *High self prediction *and return-to-work. This might be a limitation of the study. However, as none of the treatment variables predicted *Return-to-work*, we consider the potential bias achieved by the treatment as negligible.

## Conclusions

In primary-care patients with non-acute, non-specific spinal pain, including back and/or neck pain, the strong predictors of stable return-to-work were 2 socioeconomic variables, *Low total previous sick-listing *(including all diagnoses) and *Young age *(max 44 years), and 1 subjective variable, *High self prediction *(the patients' own belief in return-to-work). Objective variables from function tests and treatment variables (a programme of cognitive-behavioural rehabilitation or traditional primary care) were non-predictors. Except for *Young age*, the predictors had been insufficiently studied in previous research. Hence, our study might contribute to a widening of knowledge within clinical practice, including the allocation of treatment resources.

## Competing interests

The authors declare that they have no competing interests.

## Authors' contributions

OL was the main investigator, carried out the study, performed the analysis and drafted the manuscript. SEJ contributed to the statistical analysis. LES, as supervisor of OL, participated in all phases of the study. All authors read and approved the final manuscript.

## Pre-publication history

The pre-publication history for this paper can be accessed here:

http://www.biomedcentral.com/1471-2296/11/53/prepub
